# FALL RISK FACTORS IN COMMUNITY-DWELLING OLDER ADULTS: AN UMBRELLA REVIEW OF SYSTEMATIC REVIEWS

**DOI:** 10.1093/geroni/igae098.3365

**Published:** 2024-12-31

**Authors:** Stephanie Saunders, Cassandra D’Amore, Qiukui Hao, Ayse Kuspinar, Julie Richardson, Marla Beauchamp

**Affiliations:** McMaster University, Hamilton, Ontario, Canada; McMaster Univeristy, Hamilton, Ontario, Canada; McMaster University, Hamilton, Ontario, Canada; McMaster University, Hamilton, Ontario, Canada; McMaster University, Hamilton, Ontario, Canada; McMaster University, Hamilton, Ontario, Canada

## Abstract

Falls are an imperative public health concern, resulting in disability, mental health challenges, and increased mortality risk. An extensive body of literature has examined falls risk factors; however, it has become challenging to navigate. We summarized systematic reviews examining risk factors for falls among community dwelling older adults. We searched seven databases (Medline, Embase, CINAHL, Cochrane Library, PsychINFO, Ageline) for systematic or scoping reviews including community-dwelling older adults (≥60 years) and reporting risk factors for falls in prospective studies. Three independent reviewers screened 8,173 records and included 57 reviews. Thirty-three reviews conducted meta-analyses and 24 reported relationships from primary studies. Reviews were published between 2004-2023 and examined patients with stroke (n=2), diabetes (n=2), frailty (n=3), and cognitive impairments (n=3). Types of falls examined included recurrent falls (n=45) and injurious falls (n=18). Reviews reported risk factors across every construct of the WHO’s ICF model; health conditions (n=20 reviews), body structure and function (n=26), activity (n=23), participation (n=11), environmental factors (n=10), and personal factors (n=11). Most commonly conducted meta-analyses included: mobility impairments (demonstrating increased risk across gait, strength, and clinical performance tests, n=12/20), balance impairments (increased risk n=15/16), BMI (null risk n=12/12), frailty (increased risk n=9/10). Heterogeneity of reported effect sizes prohibited the calculation of pooled magnitudes for risk factors. Narrative synthesis results are summarized separately. Physical risk factors for falls are well established and can be addressed through interventions. Findings highlight narrow examination of psychosocial and environmental risk factors for falls in community dwelling older adults, indicating areas for future research.

